# Detecting neural assemblies in calcium imaging data

**DOI:** 10.1186/s12915-018-0606-4

**Published:** 2018-11-28

**Authors:** Jan Mölter, Lilach Avitan, Geoffrey J. Goodhill

**Affiliations:** 1 Queensland Brian Institute, The University of Queensland, Brisbane, 4072 Australia; 2 School of Mathematics and Physics, The University of Queensland, Brisbane, 4072 Australia

**Keywords:** Spontaneous activity, Population coding, Clustering

## Abstract

**Background:**

Activity in populations of neurons often takes the form of assemblies, where specific groups of neurons tend to activate at the same time. However, in calcium imaging data, reliably identifying these assemblies is a challenging problem, and the relative performance of different assembly-detection algorithms is unknown.

**Results:**

To test the performance of several recently proposed assembly-detection algorithms, we first generated large surrogate datasets of calcium imaging data with predefined assembly structures and characterised the ability of the algorithms to recover known assemblies. The algorithms we tested are based on independent component analysis (ICA), principal component analysis (Promax), similarity analysis (CORE), singular value decomposition (SVD), graph theory (SGC), and frequent item set mining (FIM-X). When applied to the simulated data and tested against parameters such as array size, number of assemblies, assembly size and overlap, and signal strength, the SGC and ICA algorithms and a modified form of the Promax algorithm performed well, while PCA-Promax and FIM-X did less well, for instance, showing a strong dependence on the size of the neural array. Notably, we identified additional analyses that can improve their importance. Next, we applied the same algorithms to a dataset of activity in the zebrafish optic tectum evoked by simple visual stimuli, and found that the SGC algorithm recovered assemblies closest to the averaged responses.

**Conclusions:**

Our findings suggest that the neural assemblies recovered from calcium imaging data can vary considerably with the choice of algorithm, but that some algorithms reliably perform better than others. This suggests that previous results using these algorithms may need to be reevaluated in this light.

**Electronic supplementary material:**

The online version of this article (10.1186/s12915-018-0606-4) contains supplementary material, which is available to authorized users.

## Background

A prominent functional property of both spontaneous and evoked neural activity is its organisation into neural assemblies [1]. Although several different meanings of the term “neural assembly” have been proposed, here we define it to mean a group of neurons whose activity tends to be coincidentally elevated, given a specific timescale for “coincident.” Such assemblies have been demonstrated in, for instance, the mammalian cortex and hippocampus [2–11] and the zebrafish optic tectum [12–15] and are believed to form a critical substrate for neural computation [3, 16–19]. Assemblies present in spontaneous activity are often similar to those driven by evoked activity [7, 12, 20, 21], and it has been suggested that this similarity increases during development as the network develops expectations about the statistics of sensorily evoked neural activity [4].

Neural population activity is sometimes recorded in the form of spikes with high temporal precision [22, 23], but more often it comes from imaging of fluorescent calcium indicators such as the GCaMP family [24, 25]. These signals can have hundreds or even thousands of dimensions and can have both high background noise and low temporal resolution. While it can be qualitatively obvious from visual inspection of the resulting time-lapse movies that neurons are organised into assemblies, quantitatively identifying and extracting these assemblies based on their firing statistics is a challenging problem. Assemblies may overlap, each neuron may be active more often than the assemblies of which it is a member, and the activity of a particular assembly may not predict with certainty the activity of every neuron it contains [15].

One starting point for extracting assemblies from a calcium imaging recording is the covariance matrix of the calcium activity. By applying principal components analysis [26], followed by either independent component analysis (ICA) [27] or Promax oblique rotation (Promax) [12] to the principal components, groups of temporally correlated neurons can be extracted. We identify two subtypes of these algorithms based on their null models for significance of the principal components, “CS” (circular shifts) and “MP” (Marčenko-Pastur) (see “Methods” section). For ICA-CS and Promax-CS significance, and the number of assemblies present was estimated using shuffling through circular random shifts, while for ICA-MP and Promax-MP this was achieved by examining eigenvalues of the covariance matrix lying outside a particular range [28]. A different starting point for the problem is to first threshold the calcium activity to a digital code, where each neuron is considered either active or not at each timestep. Frequent item set mining (FIM), which is well-established in fields of data mining such as market basket analysis, together with subsequent statistical tests (collectively FIM-X), can be used to identify groups of neurons which tend to be frequently coactive [29, 30]. Alternatively, restricting to only population activity patterns with an overall high level of activity, every set of similar patterns can be assigned a representative. These representatives can then subsequently be clustered (CORE) [7] or a singular value decomposition can be applied to a similarity map of these patterns to find significant states (SVD) [31]. A different approach based on graph theory, similarity graph clustering (SCG) [15], considers the population activity patterns as the nodes of a graph, and Bayesian statistical techniques are used to estimate the number of clusters of activity patterns present. Subsequently, spectral clustering [32] can be used to extract the clusters and, from these, reconstruct the groups of neurons that tend to be coactive.

However, for real data, it is difficult to know how well these algorithms actually work, since there is usually no way of independently verifying whether the results are accurate or not. To assess whether reliable inferences are being drawn from biological data, it is therefore critical to investigate the performance of these algorithms on surrogate data with known ground truth. Preliminary tests of the algorithms mentioned above have been performed, but mostly for spiking rather than calcium data, and only in a very limited manner, for instance using only small numbers of neurons and assemblies. It is therefore unknown how the performance of these different algorithms compares, or indeed if they perform well at all, for larger-scale, more realistic data.

Here we first investigated the performance of these algorithms on a large datasets of surrogate calcium imaging data, where performance was measured in terms of how reliably the assemblies known to be embedded were recovered. We found that SGC and ICA-CS performed very well, while Promax-MP, SVD and FIM-X generally performed poorly. For instance, Promax-MP and FIM-X showed a strong dependence on the size of the neural array. However, we also found that ICA-MP and Promax-CS could achieve performance often comparable to SGC and ICA-CS. We then applied all these algorithms to a dataset of activity in the zebrafish optic tectum evoked by simple visual stimuli, and asked whether the algorithms could recover the assemblies estimated by averaging activity patterns over presentations for each stimulus. Here the performance of SGC exceeded that of other algorithms. Overall, we therefore conclude that only some algorithms perform well for reconstructing neural assemblies from calcium imaging data.

## Results

### Generation of surrogate calcium imaging data

**Fig. 1.**
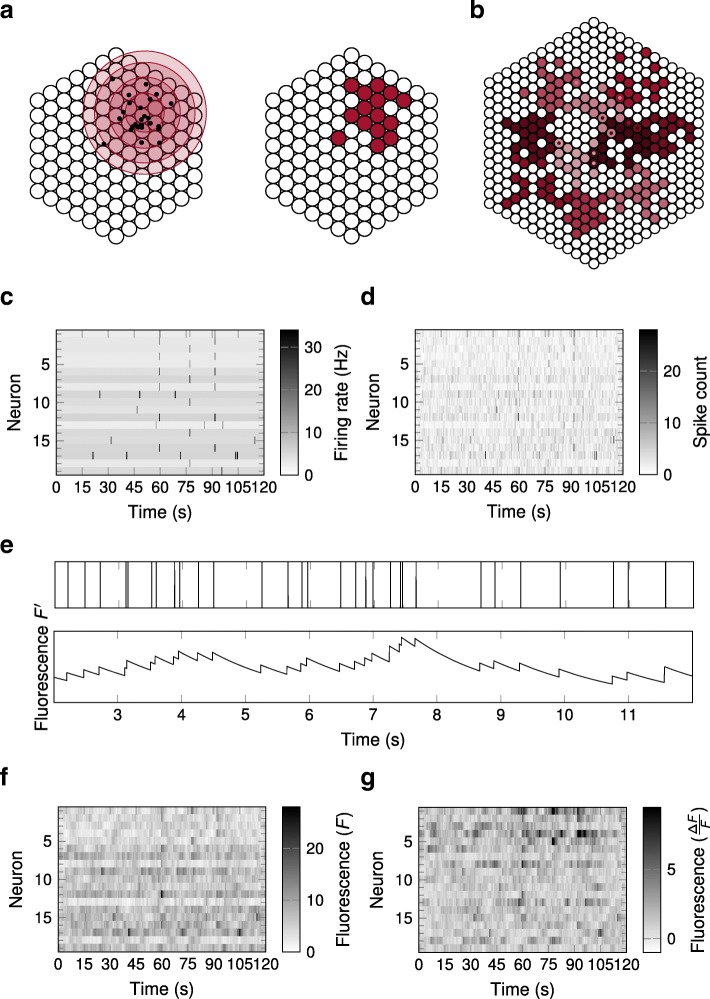
Generation of surrogate calcium imaging data. **a** On the left, points were drawn from a two-dimensional normal distribution, and a neuron was considered part of the assembly when at least one point fell within distance $\frac {1}{2}$ of its corresponding lattice point at its centre. The contour lines around the mean indicate regions of probability mass 50%, 90%, 99%, and 99.9%. The corresponding assembly is shown on the right. **b** An example of 10 assemblies (colour coded) embedded in a neural array of 469 neurons. Annuli represent neurons which overlap between two assemblies. **c** An example of the firing rates for 19 neurons over the course of 120 s. Neurons within the same assemblies simultaneously elevated their firing rates (here at 60 s, 77.5 s and 92 s). **d** The spike counts for the neurons over the course of 120 s as determined from independent Poisson random variables based on the firing rates shown in **c**. **e** The spiking events of a single neuron in a time-window of 10 s (top) and the corresponding calcium fluorescence after convolution with an exponential kernel (bottom). **f** The calcium fluorescence for the neurons over the course of 120 s. **g** The deflection of calcium fluorescence from baseline level, $\frac {\Delta F}{F}$, for the neurons over the course of 120 s. This was the signal from which the algorithms attempted to reconstruct assembly structure

The methods we used to generate surrogate data were broadly similar to previous work. To specify groups of neurons which tended to be coactive, i.e., coordinatedly increasing their firing rate, neurons were positioned on a hexagonal lattice (though spatial relationships between neurons were not taken into account in the subsequent analysis). To select the neurons in the assembly, a position in the plane was chosen at random, and then points were drawn from a two-dimensional normal distribution around this position, so that a neuron was considered part of the assembly when at least one of these random points fell into its immediate neighbourhood (see “Methods” section). Doing this multiple times allowed us to create many sets of assemblies, with a statistically controlled number of neurons per assembly and degree of overlap between assemblies for each set (Fig. 1a, b). In addition, we varied the size of the neural array while the above parameters were fixed, so that different densities of assemblies within the array were considered (see “Methods” section).

All neurons in the array were assumed to fire Poisson spikes with a fixed background rate chosen randomly for each neuron [26]. With a constant probability at each time step, each neuron elevated its rate by a factor *λ* for that time step. If this occurred for any neuron in an assembly, then all neurons in that assembly also increased their rate by a factor *λ* at that time step (Fig. 1c, d). With small probability, two assemblies could therefore be active at the same time, but this was very unlikely to occur more than once in each dataset (i.e. each simulated recording). The resulting spike trains were then convolved with an exponential calcium decay kernel with half-life of $\tau _{\frac {1}{2}}$ (Fig. 1e) [33, 34]. After applying a nonlinear saturation function noise was added to this signal from a centred normal distribution [35], and $\frac {\Delta F}{F}$ measurements were extracted from the resulting calcium traces (Fig. 1f, g) at a temporal resolution of *ΔT*. Notation and default parameter values are summarised in Table 1. Unless otherwise indicated, graphs shown in the subsequent figures represent performance variations as individual parameters were varied from this default set.

**Table 1:** Table of default parameter values used to generate surrogate calcium imaging datasets

Parameter	Default value	Variation range
Size of neural array	469 neurons	217–919 neurons
Number of embedded assemblies (*k*)	10	1–20
Mean assembly size	16 neurons	6–28 neurons
Assembly overlap	[0,0.05]	0.05–0.35
Simulation duration (*T*)	3600 s	0–7200 s
Temporal resolution (*ΔT*)	500 ms	100–500 ms
Spike time temporal resolution (*δT*)	1 ms	—
Calcium indicator half-life ($\tau _{\frac {1}{2}}$)	1 s	0–2 s
Saturation constant (*κ*)	*∞*	0–1000
Background firing rate (*R*)	[1,6] Hz	—
Event duration (*ΔT*^∗^)	500 ms	—
Event frequency (*f*^∗^)	10 mHz	0–10 mHz
Event firing rate multiplier (*λ*)	6	1–25
Standard deviation of Gaussian noise (*σ*)	0	0–8

**Fig. 2.**
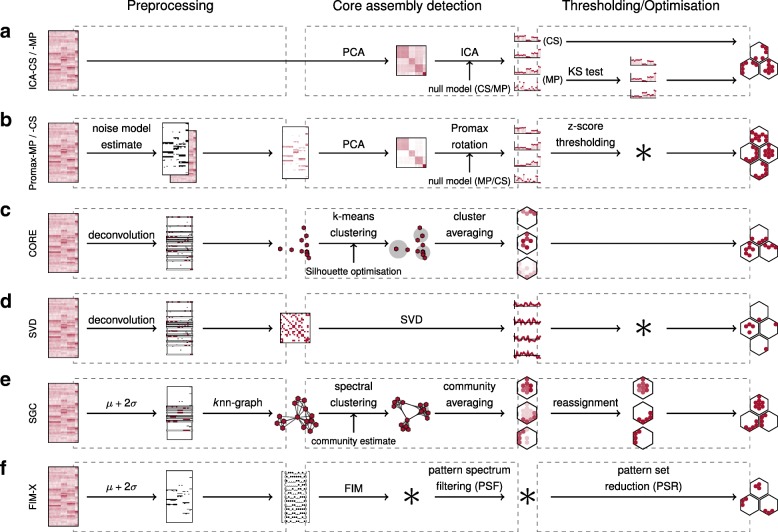
Schematics of the different algorithms investigated All algorithms can be divided into three phases: preprocessing, core assembly detection and thresholding/optimisation. **a** In the ICA algorithms, PCA is applied to $\frac {\Delta F}{F}$, followed by ICA to the significant principal components. The null model for significance is either determined from circular shifts (ICA-CS) or given as the Marčenko-Pastur distribution (ICA-MP). The resulting principal components are either thresholded directly (ICA-CS) or after a KS test (ICA-MP) in order to arrive at the assemblies. **b** In the Promax algorithms, $\frac {\Delta F}{F}$ is first reduced to its significant calcium transients, before PCA is applied. The null model for significant principal components is either given as the Marčenko-Pastur distribution (Promax-MP) or determined from circular shifts (Promax-CS). These principal components are rotated by means of Promax before z-score thresholding the components to arrive at the assemblies. **c** In the CORE algorithm, $\frac {\Delta F}{F}$ is deconvolved and the resulting spike probabilities are thresholded into a binary signal. The activity patterns with a high level of activity are reduced to a set of core activity patterns (or ensembles) which are clustered using k-means clustering and the activity patterns of every community are averaged to arrive at the assemblies. **d** In the SVD algorithm, $\frac {\Delta F}{F}$ is deconvolved and the resulting spike probabilities are thresholded into a binary signal. From the activity patterns with a high level of activity a similarity map is constructed and thresholded before SVD is applied. The assemblies were then inferred from the activity patterns corresponding to every significant singular vector. **e** In the SGC algorithm, $\frac {\Delta F}{F}$ is thresholded to a binary signal and the activity patterns with a high level of activity are arranged into a graph according to their similarity. The graph is split into its communities using spectral clustering and the activity patterns of every community are averaged to arrive at the assemblies. **f** In the FIM-X algorithm, $\frac {\Delta F}{F}$ is thresholded into a binary signal and FIM is applied to find co-active neurons as frequent item sets. These frequent item sets are reduced by PSF and PSR involving some additional statistical tests to arrive at the assemblies

Eight different algorithms were used to reconstruct the embedded assemblies (Fig. 2). For most algorithms, the implementations were either publicly available or kindly given to us by the original authors. Four algorithms considered the neuron-neuron correlation, applying PCA either in connection with ICA (ICA-CS) [26, 27] or Promax oblique rotations (Promax-MP) [12, 36], and we also investigated a novel variant of ICA-CS (ICA-MP), as well as a novel variant of Promax-MP (Promax-CS) (see “Methods” section). Instead of looking at neuron-neuron correlation, the remaining four algorithms rather considered the relation between different states of the whole population. One such algorithm involved applying frequent item set mining in conjunction with additional statistical tests (FIM-X) [29, 30]. The CORE algorithm considers the similarity between activity patterns and defined a set of core representatives which were then subsequently clustered [7], while another algorithm considered a similarity map of activity patterns to which SVD [31] was applied. Finally, the algorithm which we refer to as “similarity graph clustering” (SGC) represented the similarity of activity patterns on a graph and clustered them within this graph [15].

The performance of the different algorithms was measured by the match between the number of assemblies found versus the number embedded, and the similarity of the embedded and the reconstructed assemblies. The latter was quantified with a “Best Match” score [37] (see “Methods” section). Some of the algorithms tended to overestimate the number of assemblies, which consequently reduced the performance as measured by the Best Match score even if some of the recovered assemblies were similar to the embedded ones. As a more generous estimate of performance, we therefore also considered an “optimised Best Match” score (Additional file 2), where we measured the similarity between the embedded assemblies and the subset of recovered assemblies which most closely matched these (see “Methods” section).

### Performance with varying array size, number of assemblies, assembly size and overlap

**Fig. 3.**
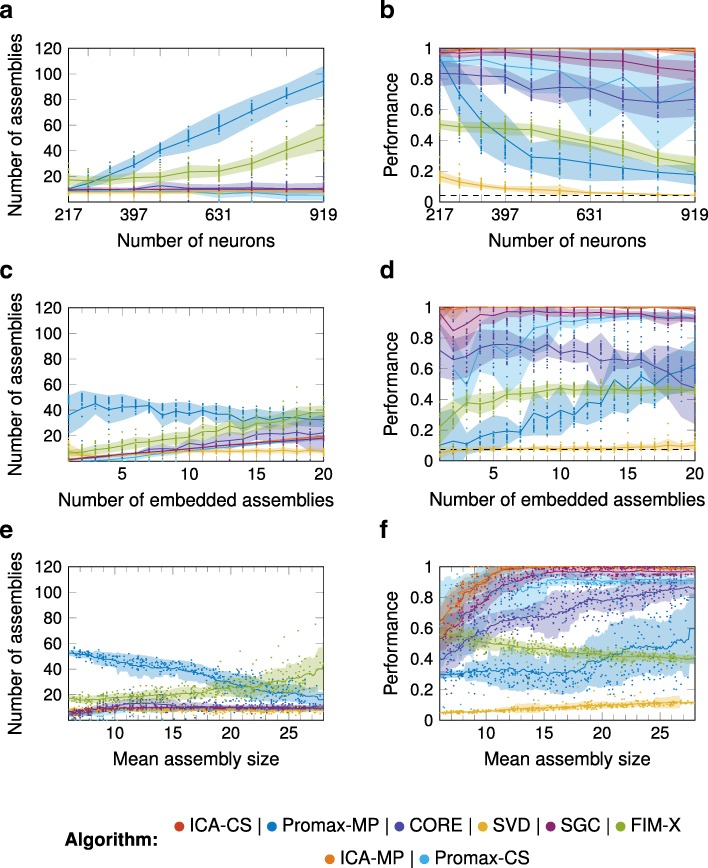
Performance as a function of size of the neural array, number of embedded assemblies, and assembly size In every graph and for every algorithm the mean is depicted by a solid line together with the region of one standard deviation above and below. In the graphs for the performance, measured in terms of the Best Match score, the black dashed line indicates chance level of the Best Match score. **a, b** Varying the size of the neural array. With an increasing size of the neural array, Promax-MP and FIM-X detected an increasing number of assemblies and, consequently, their performance decreased. ICA-CS, ICA-MP, SGC, CORE, and SVD detected a constant number of assemblies and except for SVD showed good performance. Promax-CS performed well for smaller neural arrays, but slightly underestimated the number of assemblies in larger arrays. **c, d** Varying the number of embedded assemblies. Promax-MP detected an almost constant number of assemblies. ICA-CS, ICA-MP, SGC, Promax-CS, and FIM-X detected an increasing number of assemblies as the number of embedded assemblies increased, although FIM-X overestimated the total number. When there were only few assemblies embedded, Promax-CS underestimated the total number, while when there were more assemblies embedded, CORE overestimated and SVD underestimated the total number. **e, f** Varying the assembly sizes. ICA-CS, ICA-MP, SGC, Promax-CS, CORE, and SVD detected a constant number of assemblies except when the embedded assemblies were particularly small. Promax-MP and FIM-X overestimated the number of assemblies

We first investigated how the number of assemblies detected by the algorithms varied with the size of the neural array. Some neurons may not participate in any assembly, and instead, their activity provides only noise. We embedded 10 assemblies into an array of size varying from 217 to 919 neurons. SGC, ICA-CS, ICA-MP, CORE, and SVD recovered the correct number of assemblies (Fig. 3a, b; Additional file 2), though the performance of SVD (i.e. match of recovered assemblies to true assemblies) was low. For Promax-MP, the performance was good when the neural array was small. While Promax-CS also found the correct assemblies for small neural arrays, for large arrays, it slightly underestimated the number of assemblies.

Next, we tested how the number of assemblies detected varied with the number of assemblies embedded. Again SGC, ICA-CS, ICA-MP, and to some extent Promax-CS performed well, but Promax-MP, FIM-X, and CORE found an excess and of assemblies and SVD underestimated the total number (Fig. 3c, d; Additional file 2). We then varied the mean assembly size for 10 embedded assemblies (Fig. 3e, f; Additional file 2). Again, Promax-MP and FIM-X did not perform well for any assembly size. The performance of SGC, ICA-CS, ICA-MP, Promax-CS, and CORE was good except for small assembly sizes, where the performance of SGC, ICA-CS, and ICA-MP was similar to that of FIM-X. The performance of all algorithms decreased with an increasing degree of overlap in the assemblies (Additional file 1 : Figure S1A,B; Additional file 2).

In summary, we found that the size of the neural array strongly affects the performance of Promax-MP and FIM-X. In contrast, SGC, ICA-CS, and ICA-MP as well as Promax-CS detected the correct number of assemblies irrespective of how many were embedded, and their performance was generally better for larger assemblies. The performance of SVD was overall low despite recovering the correct number of assemblies.

### Performance with varying signal strength

We then investigated how the performance of the algorithms varied with the strength of the signal compared to the noise. Signal strength is controlled by several factors including the number of assembly events present (determined by the length of the simulation and the event frequency), the temporal resolution of the calcium signal, the calcium indicator’s decay time, the saturation of the calcium indicator, the firing rate multiplier during assembly events, and additional noise added to the calcium signal.

**Fig. 4.**
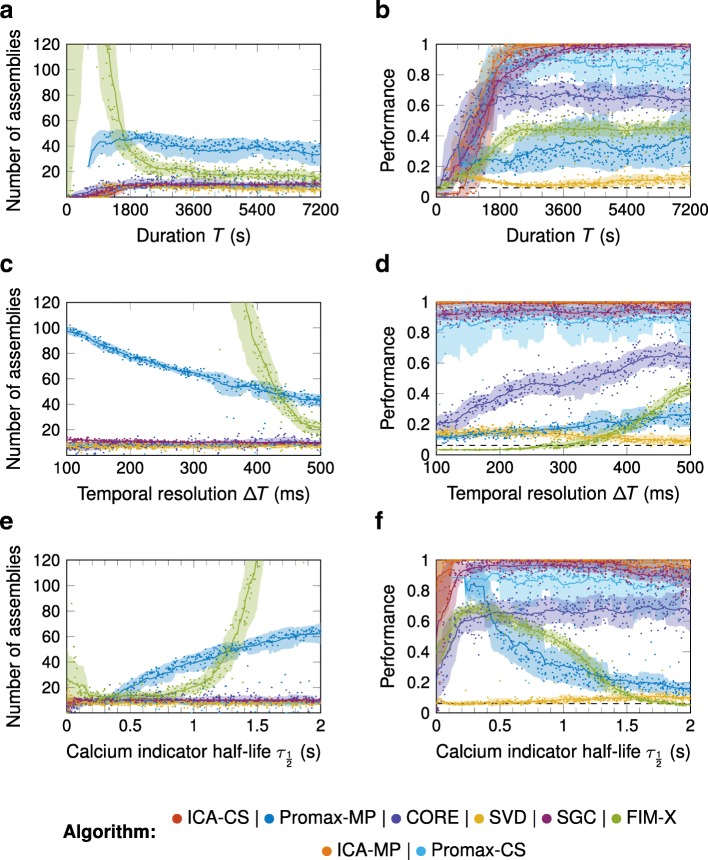
Performance as a function of simulation duration, temporal resolution, and calcium indicator half-life Graphing conventions as in Fig. 3. **a**, **b** Varying the simulation duration *T*. With increasing *T*, the performance of ICA-CS, ICA-MP, SGC and Promax-CS increased and they detected a constant number of assemblies beyond *T*=1800s. Promax-MP overestimated the total number, and the number detected by FIM-X showed a steep peak, while SVD underestimated the total number. **c**, **d** Varying the temporal resolution *ΔT*. With increasing *ΔT*, Promax-MP and FIM-X detected a decreasing number of assemblies. They both overestimated the total number, particularly FIM-X at small *ΔT*. SVD underestimated the total number of assemblies. ICA-CS, ICA-MP, SGC, Promax-CS and CORE detected a constant number of assemblies and ICA-CS, ICA-MP, SGC and Promax-CS also showed good performance. **e**, **f** Varying the calcium indicator half-life $\tau _{\frac {1}{2}}$. With increasing $\tau _{\frac {1}{2}}$ Promax-MP and FIM-X detected an increasing number of assemblies. ICA-CS, ICA-MP, SGC, Promax-CS, CORE and SVD detected a constant number of assemblies and ICA-CS, ICA-MP, SGC, Promax-CS and CORE showed good performance

As expected, the performance for all algorithms increased with simulation duration and therefore the average absolute number of assembly events. This increase was slow for Promax-MP and FIM-X, but relatively rapid for SGC, ICA-CS, ICA-MP, Promax-CS, and CORE, which apart from CORE eventually reached saturating performance for the default parameter set (Fig. 4a, b; Additional file 2). Surprisingly, the number of detected assemblies for FIM-X peaked and then declined. Greater temporal resolution of the calcium signal had no effect on the performance of SGC, ICA-CS, ICA-MP, and Promax-CS, which was overall good, but decreased the performance of Promax-MP and FIM-X, apparently because they found increasing numbers of assemblies as more data was available. For CORE, the absolute number of assemblies was correct, while SVD underestimated it (Fig. 4c, d; Additional file 2).

While results for SGC, ICA-CS, ICA-MP, Promax-CS, CORE, and SVD were quite robust to calcium indicator half-life $\tau _{\frac {1}{2}}$, the performance of Promax-MP and FIM-X decreased as $\tau _{\frac {1}{2}}$ increased (Fig. 4e, f; Additional file 2). However, for very low $\tau _{\frac {1}{2}}$ neither Promax-MP or Promax-CS returned results because their noise model was unable to fit the data.

**Fig. 5.**
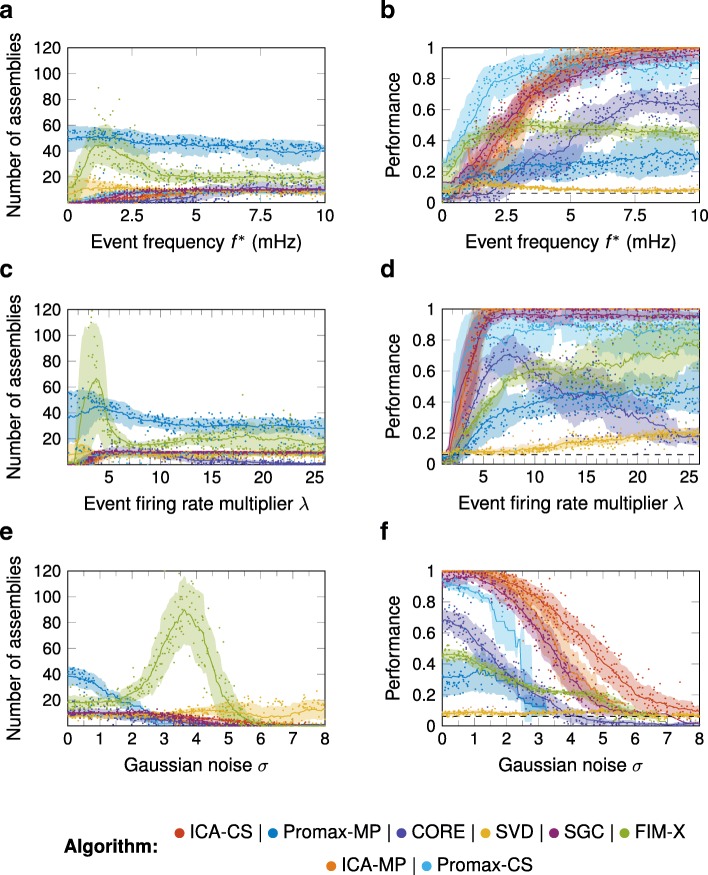
Performance as a function of the event frequency, event firing rate multiplier, and the standard deviation of the noise Graphing conventions as in Fig. 3. **a**, **b** Varying the event frequency *f*^∗^. With increasing *f*^∗^, the performance of ICA-CS, ICA-MP, SGC, Promax-CS and CORE increased and they detected a constant number of assemblies beyond *f*^∗^=5mHz. At low frequencies the performance of Promax-CS exceeded that of ICA-CS, ICA-MP and SGC. Promax-MP and FIM-X both overestimated the number of assemblies. **c**, **d** Varying the event firing rate multiplier *λ*. With increasing *λ*, the performance of ICA-CS, ICA-MP, SGC, and Promax-CS increased and, when *λ* exceeded about 4, they detected a constant number of assemblies and showed good performance. Promax-MP and FIM-X both overestimated the number of assemblies. The performance of CORE first increased but then consequently decreased as it underestimated the total number of assemblies. **e**, **f** Varying the standard deviation *σ* of the Gaussian noise. With increasing *σ*, every algorithm (except FIM-X, which instead showed a peak, and SVD) detected a decreasing number of assemblies and, consequently, their performance decreased. For noise levels beyond *σ*=3 neither Promax-MP or Promax-CS yielded any results since they were not able to fit the noise model to the data

The performance of all algorithms increased with event frequency, but much faster for SGC, ICA-CS, ICA-MP, and Promax-CS than Promax-MP, FIM-X, CORE, and SVD (Fig. 5a, b; Additional file 2). As for the simulation duration, variations in the event frequency change the average number of repetitions for every assembly event. However, the noise activity between events differs between these scenarios, so that simulation duration and event frequency are not interchangeable parameters (Additional file 3). The event firing rate multiplier *λ* determined the increase in firing rate when a neuron was active, and thus how clearly distinguishable such an event was in terms of the increase in fluorescence from the background. As *λ* increased, SGC, ICA-CS, ICA-MP, and Promax-CS showed a threshold at about *λ*=2 (for the default set of other parameters) below which no assemblies were detected, and beyond which the performance increased close to optimal (Fig. 5c, d; Additional file 2). In contrast, the performance of Promax-MP and FIM-X increased very slowly with *λ*, and again, the number of assemblies found by FIM-X showed a peak and then declined. As for SGC, ICA-CS, ICA-MP, and Promax-CS, the number of detected assemblies and performance first increased, but then decreased again.

As the noise parameter *σ* added to the calcium signal increased, the performance of SGC, ICA-CS, and ICA-MP remained close to 1 until about *σ*=2, beyond which their performance declined slowly (Fig. 5e, f; Additional file 2). The same behaviour could be seen in the performance of CORE, however, at a lower value. The performance of Promax-CS dropped more rapidly with *σ*, and neither Promax-MP, FIM-X, nor SVD performed well for any value of *σ*.

In reality, calcium signals saturate. We therefore also considered the effect of a simple nonlinear saturation in the calcium signal with saturation constant *κ* [33–35]. However, this had very little effect on the performance of any of the algorithms (Additional files 1 and 2).

In summary, for all algorithms, the performance increased with either higher event frequency or longer simulation durations. Performance remained constant for SGC, ICA-CS, and ICA-MP, Promax-CS, as well as CORE when the temporal resolution was increased, while for Promax-MP and FIM-X it decreased. The calcium indicator half-life did not affect the performance of SGC, ICA-CS, ICA-MP, Promax-CS, or CORE. Furthermore, performance increased with greater event firing rate multiplier for all algorithms, although SGC, ICA-CS and ICA-MP reached peak performance faster. Again, performance of SVD was low despite recovering the correct number of assemblies.

### Application to real data

**Fig. 6.**
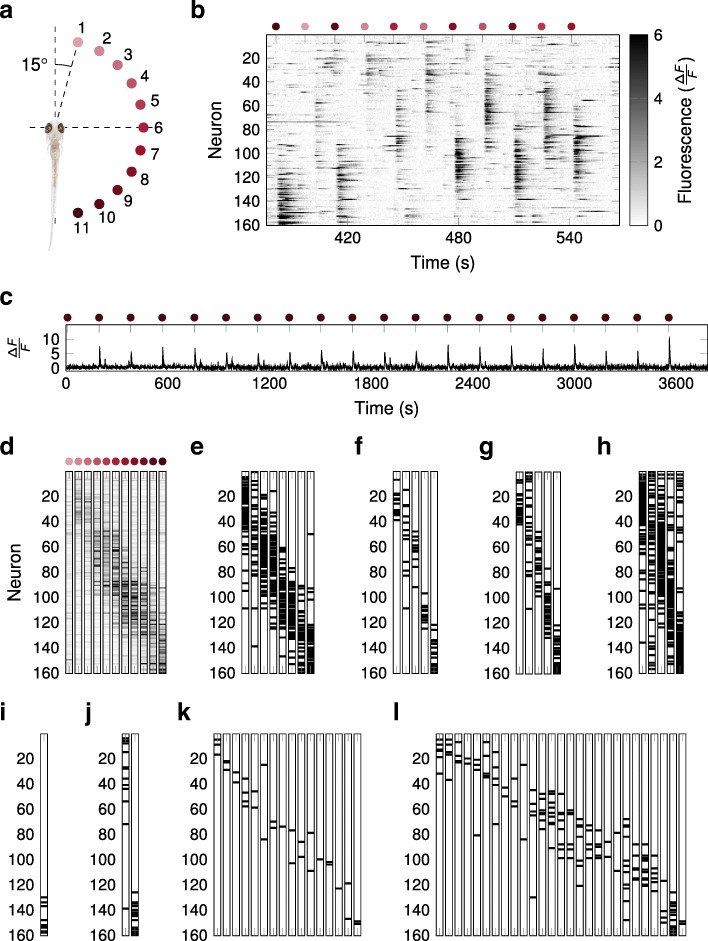
Application of the different algorithms to stimulus-evoked calcium imaging data from the larval zebrafish optic tectum. **a** 11 different stimuli were shown to the fish. The stimuli were separated by 15° in the visual field of the fish. **b** The deflection of calcium fluorescence from baseline level, $\frac {\Delta F}{F}$, for the 160 neurons over about 180 s of the recording. The neurons are ordered by their anterior-posterior position in the tectum. The stimuli were presented in the order 11 – 1 – 10 – 2 – 6 – 3 – 8 – 4 – 9 – 5 – 7 as indicated. **c** Example calcium trace over the course of the whole experiment from a neuron particularly responsive to stimulus 11, whose onset is indicated. The overall noise is relatively low and the peaks in fluorescence are clearly visible. **d** The average population response in terms of fluorescence ($\frac {\Delta F}{F}$) to the 11 different stimuli. The responses to the first 3 stimuli were weak compared to the others. **e–j** Graphical representations of the assemblies recovered by the different algorithms. The neurons which were part of the respective assemblies are marked in black. **e** SGC recovered 8 assemblies. **f** ICA-CS recovered 5 assemblies. **g** Promax-CS recovered 5 assemblies. **h** SVD recovered 5 assemblies. **i** CORE recovered 1 assembly. **j** ICA-MP recovered 2 assemblies. **k** Promax-MP recovered 16 assemblies. **l** FIM-X recovered 27 assemblies

We then tested the performance of the algorithms on a dataset of stimulus-evoked activity in the zebrafish optic tectum. Eleven different stimuli were shown to the fish via a projector in the form of small spots separated by 15° in the visual field (Fig. 6a). The responses to these stimuli were clearly visible within the population activity (Fig. 6b). $\frac {\Delta F}{F}$ values were much larger for the spot presentations than for the intervening periods of spontaneous activity, and this contrast was also much more pronounced than in our simulated data with injected assemblies (Fig. 6c; cf. Fig. 1). We estimated a reference assembly configuration from the average activity evoked by each stimulus over 20 repetitions, and then asked if the algorithms would find these assemblies. A neuron was regarded as part of an assembly if it was, on average, substantially more active in response to the corresponding stimulus than across all stimuli (see “Methods” section). Although tectal responses were seen for all the stimuli, the activity evoked by stimuli 1–3 was weaker and more overlapping (Fig. 6d). Thus we expected that between 8 and 11 assemblies should be found for these data.

All algorithms found sets of assemblies which were appropriately localised, and preserved topographic ordering in the optic tectum (Fig. 6e–l). SGC found 8 and SVD 5 relatively dense assemblies (Fig. 6e, h). For the other algorithms, there was a large diversity in the sparseness and the number of the assemblies they found, ranging from 1 for CORE (Fig. 6i) to 27 for FIM-X (Fig. 6l). Compared to the reference configuration as defined above, either some assemblies appear to have been missed or subdivided to produce a large number of sparse assemblies.

Qualitatively, Fig. 6 suggests that SGC gave the most accurate results, followed by Promax-CS and then ICA-CS. We confirmed this quantitatively by calculating the Best Match score with respect to the estimated reference assembly configuration (Additional file 4). This suggests that SGC was the best algorithm for reconstructing assemblies defined by evoked activity in real data.

## Discussion

We have shown that assembly detection algorithms can produce very divergent results on the same set of calcium imaging data (real or surrogate), in terms of both the number and neural identity of the assemblies found. In general, SGC and ICA-CS performed very well and were quite robust to the number and overlap of assemblies, the size of the array, and the temporal resolution. By making novel modifications to ICA-CS (i.e. ICA-MP) and Promax-MP (i.e. Promax-CS), we found that these algorithms could also perform very well on a wide range of surrogate data, with ICA-MP being much faster than ICA-CS. However, SGC still produced the best match for real data. There were big differences in the computational requirements of the algorithms (Additional file 5), with the success of SGC coming at the cost of one to three orders of magnitude more runtime than other algorithms. However, in future work, the SGC algorithm could potentially be parallelised to reduce this cost. An important observation was that performance of all algorithms could depend strongly on the parameters of the data (e.g. duration and event frequency), suggesting caution in attempting to extract assemblies from data which may not be well suited to such analysis.

The original Promax-MP algorithm relies on comparing the eigenvalues of the covariance matrix with the null distribution expected for a random matrix (Marčenko-Pastur), with the eigenvectors associated with the eigenvalues falling outside the support of the null distribution providing the assemblies [28]. However, the data we have investigated here is unlikely to satisfy the assumptions required for this procedure to work well, hence explaining the algorithm’s relatively poor performance. In particular, for the Marčenko-Pastur distribution to be a valid null distribution, independence within the fluorescence time series is assumed. However, due to the slow dynamics of the calcium indicator, this assumption is not met in the context of calcium imaging. If there are any temporal auto-correlations in the signals of the single neurons, the threshold on the significant correlations derived from the Marčenko-Pastur distribution will always overestimate the true number of assemblies. Moreover, the number of these correlations increase with the size of the neural array. However, this effect can be compensated by applying further statistical tests as we have demonstrated in case of the ICA-MP, where the algorithm was then able to produce results almost as good as ICA-CS but at a much lower computational cost.

The algorithms we have investigated in this study can be divided into two classes, with ICA-CS, ICA-MP, Promax-MP and Promax-CS in one class and SGC, CORE, SVD and FIM-X in the other. The algorithms in the first class are all based on the application of PCA to the pairwise correlations between neurons. Since the principal components are necessarily orthogonal, PCA alone will generally yield disjoint assemblies. In order to overcome this limitation, these methods further transform the principal components by means of ICA or Promax oblique rotation. The algorithms in the second class instead consider binary patterns of activity in a population. From that perspective, these algorithms can then be broadly described as performing some form of clustering of activity patterns, without restricting the shape of the assemblies.

However, the overarching problem in all algorithms, besides distributing neurons into the assemblies, is to estimate the number of assemblies in the first place. The impact of this estimate can be seen in the comparison between Promax-MP and Promax-CS. As discussed above, the only difference between these two algorithms is the evaluation of the significance of principal components, which translates into an estimate for the number of assemblies. The SGC algorithm instead expends great computational effort to find an estimate for the number of assemblies. The same is true for the FIM-X algorithm, though this was less successful in the context studied here.

With the ICA-MP and the Promax-CS algorithms, we have introduced slight variations which offer improvements over their original versions: ICA-MP is more computationally efficient than the ICA-CS algorithm, and Promax-CS detects assemblies better than the Promax-MP algorithm. In principal, there are many more algorithmic variations that could be obtained by combining components from different algorithms to create new variants, and some of these could potentially improve performance in terms of computational efficiency and/or assembly detection. However, our focus here has been primarily on comparing algorithms in the form in which they were originally proposed.

It is important to note that here we have only investigated calcium imaging data, which can be quite different from electrophysiology data. Indeed FIM-X was originally designed for the latter and has not previously been applied to calcium imaging data. In particular, we noticed that assemblies found by FIM-X tended to be sparse. This was a consequence of the sporadic activity of single neurons and the fact that an assembly is required to be active as a whole at every instance in order to be detected in this algorithm. The normally high temporal resolution of electrophysiology data means that many interesting questions can then be asked about patterns of sequential activation between neurons [38–42], which are normally not addressed in the context of calcium imaging where the temporal resolution is limited by the calcium indicators. How such temporal patterns could be detected has been addressed by several different methods [43–47]. The idea between one of these [47] is quite similar to the SGC algorithm: After defining a notion of distance between sequences of activity patterns, these are clustered in a feature space, from which the assemblies are then reconstructed.

A recent development of FIM-X is the SPADE algorithm [46], which is intended to be applicable to both spiking and calcium imaging data. We attempted to include this algorithm in our study based on code kindly provided by the authors, but unfortunately the runtime proved too long for our test datasets. In general, we restricted our consideration to algorithms for which robust code was already available. However, the code we are making available with this paper provide the opportunity to test the performance of any other algorithm in the future.

In summary, each algorithm has advantages and disadvantages. The ICA and Promax algorithms are computationally very fast but correctly estimating the number of assemblies tends to be a challenge, although this can be alleviated in the CS variants. In addition, these algorithms implicitly assume the assemblies are non-overlapping, though later steps relax this condition. The CORE algorithm embraces shuffling as a means of model-free estimating the significance of various correlations in the data. However, in particular for large datasets, this makes the algorithm also computationally slow. The FIM-X algorithm is based on well-established algorithms, but is quite sensitive to noise. The SGC algorithm provides good estimates for the number of assemblies with minimal assumptions about their shape and relation to each other. However, its runtime is at least an order of magnitude greater than any other algorithm, and three orders of magnitude greater than ICA-MP. On the other hand, unlike most other algorithms, its runtime does not increase with the number of neurons.

## Conclusions

Overall, our study demonstrated that the detection of neural assemblies varies considerably with the choice of algorithm. For real data, each experiment may occupy a slightly different region of parameter space, and low Best Match scores do not necessarily imply that any general qualitative inferences drawn from the biological data are incorrect. However, our findings do argue that previous results using these algorithms may need to be reevaluated in this light.

## Methods

### Neural arrays and generation of sets of random assemblies

To arrange a set of neurons in a plane, we used a hexagonal lattice of unit spacing, in which a neuron was placed at every lattice point. An assembly was generated by drawing points from a two-dimensional normal distribution with mean *μ*, standard deviation *σ* and corresponding isotropic covariance matrix *σ*^2^*I* (where *I* is the identity matrix). The centre *μ* was drawn from a uniform distribution on a ball around the origin. A neuron in the array was then considered part of the assembly when at least one of the points drawn from the two-dimensional normal distribution fell within distance $\frac {1}{2}$ of its corresponding lattice point in the hexagonal neural array. When considering sets of many assemblies, we controlled the overlap between the assemblies in terms of the mean pairwise Szymkiewicz-Simpson coefficient, defined as $\frac {|{A \cap A'}|}{\min \{|{A}|,|A'|\}}$ for two sets *A* and *A*^′^ (*A*∪*A*^′^ and *A*∩*A*^′^ denote the union and intersection of *A* and *A*^′^ respectively, and | · | for a set is its cardinality), by varying the radius of the region from which the assembly centres were drawn.

### Simulation of population calcium fluorescence activity

Calcium fluorescence activity was simulated in two steps. The first step was to assign firing rates. Every neuron in the array was assigned a background firing rate independently and uniformly from the range *R*, so that for neuron *n* the firing rate was $r^{(n)}(t) = r_{0}^{(n)}$, constant for $t = 1, \ldots \lceil {\frac {T}{\Delta T}}\rceil $ (we denote by ⌈ · ⌉ and ⌊ · ⌋ the ceiling and floor functions respectively). At each time *t*, every neuron *n* had a probability *f*^∗^*ΔT* of increasing its firing rate by a factor of *λ* so that $r^{(n)}(t) = \lambda r_{0}^{(n)}$; however, for neurons within an assembly, this increase in firing rate was coordinated across all neurons of the assembly. Hence, given the collection of assemblies $\mathcal {A}$, we selected the events for every assembly $A \in \mathcal {A}$ independently and increased the firing rate at these times for every unit *n*∈*A*, after which we proceeded separately for every neuron which was not part of any assembly (Fig. 1c). In order to realise a fixed event duration *ΔT*^∗^ irrespective of the duration of a time step *ΔT*, given the times *t* for an event, we extended it to the times $t + 1, \ldots t + \lfloor {\frac {\Delta T^{*}}{\Delta T}}\rfloor - 1$ and in addition with probability $\frac {\Delta T^{*}}{\Delta T} - \lfloor {\frac {\Delta T^{*}}{\Delta T}}\rfloor \in \left [ 0, 1 \right [$ to the time $t + \lfloor {\frac {\Delta T^{*}}{\Delta T}}\rfloor $ independently for every event and unit. The spike counts at every time interval *t* and for every unit *n*, *s*^(*n*)^(*t*), were then determined from independent Poisson random variables with mean *r*^(*n*)^(*t*)*ΔT* (Fig. 1d) [26].

The second step was to generate spikes and the corresponding calcium fluorescence signal. Every time interval *t* for every unit *n* was expanded into spike-time intervals *δT*, and *s*^(*n*)^(*t*) of them were selected independently for a spike to occur within. This resulted in a spike sequence of length $\frac {T}{\delta T}$ for every unit *n*, *z*^(*n*)^, where for $t = 1, \ldots \frac {T}{\delta T}$ *z*^(*n*)^(*t*)=1 if there is a spike in the time interval and *z*^(*n*)^(*t*)=0 otherwise, from which we obtained the calcium fluorescence signal by a discrete convolution with an exponential kernel with a half-life time of $\tau _{\frac {1}{2}}$ and subsequent application of a nonlinear saturation function [33–35]. We then added noise from a centred normal distribution. More precisely, the calcium fluorescence signal of unit *n* at time resolution *δT* was given as

$${{\begin{aligned} \hspace{15pt} {F'}^{(n)}(t) \,=\, &S_{\kappa}\left({\sum_{t' = 0}^{\lceil{2 \log_{2}(10) \frac{\tau_{1/2}}{\delta T}}\rceil } z^{(n)}(t - t')\mathrm{e}^{-\ln(2) \frac{t' \delta T}{\tau_{1/2}} } }\right) \!\\&+ \mathcal{N}_{0,\sigma}^{(n)}(t) \quad \!\!\!\!\!\!\!\text{for}\ t \,=\, 1, \ldots \frac{T}{\delta T} \end{aligned}}} $$

where it was assumed that *z*^(*n*)^(*t*−*t*^′^)=0 whenever *t*−*t*^′^<1, $S_{\kappa }(x) = \kappa \frac {x}{x + \kappa }$ is a nonlinear saturation function, where *S*_*κ*_(*x*)=*x* for *κ*=*∞*, and $\mathcal {N}_{0,\sigma }^{(n)}(t)$ a sequence of independent and normally distributed random variables with mean 0 and standard deviation *σ*.

In order to obtain the calcium fluorescence signal at time resolution, *ΔT* this calcium fluorescence signal was downsampled in steps of $\frac {\Delta T}{\delta T}$, so that the (downsampled) calcium fluorescence signal for neuron *n* was

$$F^{(n)}(t) = {F'}^{(n)}\left(\frac{\Delta T}{\delta T} t\right) \quad \text{for} t = 1, \ldots \frac{T}{\Delta T}{.} $$

Generally, when simulating this calcium fluorescence activity, we extended the duration of the simulation by an offset time in order for the calcium activity to build up. We chose this time to be twice the calcium indicator’s half-life. This offset was later removed from the calcium fluorescence signal.

From the calcium fluorescence signal, the deflection from the baseline fluorescence level, $\frac {\Delta F}{F}^{(n)}$ for neuron *n*, was determined, where the baseline fluorescence was fit using a Kalman smoother with a width factor of $\frac {{15}\ \mathrm {s}}{\Delta T}$ and a Gaussian noise model [48].

This model for generating calcium fluorescence signals is relatively simple and does not consider a number of additional potential sources of noise. These include mixing of signals from neighbouring cells due to limited spatial resolution, noise induced by the neuropil in mammalian cortex, and non-uniform expression of the fluorescent indicator between different cells. However, we show that some algorithms already do not perform well even without taking into account these additional noise sources.

### Application of the different algorithms

#### ICA-CS algorithm

In its original form, the ICA-CS algorithm [26, 27], whose implementation was kindly made available to us by Vítor Lopes-dos-Santos, started with a set of spike trains, which were then binned at a temporal resolution *ΔT* so that the objects under consideration were effectively the spike counts (z-score normalised per neuron). These spike counts were arranged in a *N*×*P* matrix *X* with *N* the number of neurons and *P* the number of time-bins, where due to the z-score normalisation the rows of *X* had zero mean and unit variance. The eigenvalue distribution of the corresponding auto-correlation matrix $\frac {1}{P} XX^{T}$ was then compared with a null distribution. This null distribution was obtained by means of circular random shifts [27], where for every neuron independently the spike counts were circularly shifted by a random offset. Thus, while correlations between neurons were destroyed, temporal correlations for each neuron were preserved. Iterating the circular random shifts, the null distribution was estimated as the average eigenvalue distribution from 500 rounds, and eigenvalues exceeding the 95th percentile of this null distribution were then assumed to be due to correlations between neurons in the data. In particular, the eigenvectors (principal components) corresponding to these eigenvalues were assumed to represent the assemblies. These principal components were then rotated by means of ICA [49] in 500 iterations of the fastICA algorithm in order to find the ideal assembly vectors in which every neuron’s component was interpreted as corresponding to its affinity to the respective assembly. The assembly was defined as the neurons with particularly strong affinity, determined by thresholding the absolute values of the components at two standard deviations above the mean for that vector. Applying this method to calcium activity data, we used $\frac {\Delta F}{F}$ as a proxy for the actual spike counts or firing rates.

#### ICA-MP algorithm

Instead of using circular shifts, the significant principal components could alternatively be estimated employing the Marčenko-Pastur distribution [28, 50] to compare with the observed eigenvalue distribution. The Marčenko-Pastur distribution is the limit of the empirical spectral density of an auto-correlation matrix, if all its components were independent. Hence, eigenvalues outside of the compact support of this distribution were then assumed to be due to correlations in the data. Computationally, this was more efficient than estimating the null distribution from circular random shifts. However, close inspection of the assembly vectors found revealed those among them where no neurons had a particularly large affinity to the assembly, resulting in the detection of spurious assemblies. To address this problem, we discriminated the assembly vectors before the thresholding using a one-sample Kolmogorov-Smirnov (KS) test on the z-scored components at a significance level of *α*=10^−10^ (determined empirically), and considered only those assembly vectors that were rejected given the null hypothesis of a standard normal distribution. From the remaining assembly vectors, we reconstructed the assemblies as before.

#### Promax-MP algorithm

The Promax-MP algorithm [12], similar to the ICA algorithms above, aimed to detect significant correlations between neurons using PCA, but with adaptations for calcium imaging data and some further statistical tests. The implementation, as part of a toolbox for the analysis of calcium imaging data, is available at https://github.com/zebrain-lab/Toolbox-Romano-et-al [51].

Instead of applying principal component analysis directly to the calcium fluorescence signals, first a noise model was fitted. Calcium transients which differed significantly from this noise signal were then extracted and the calcium signals around the significant transients were set to 0. As above for the ICA-MP algorithm, significant principal components of the auto-correlation matrix were determined by means of the Marčenko-Pastur distribution but including the Tracy-Widom correction to account for finite size effects. The significant principal components were rotated by means of the Promax algorithm [52], in order to concentrate the principal component loadings along them. Lastly, neurons were assigned to assemblies if their z-score normalised vector component along the respective rotated principal component exceeded a threshold determined from the data.

Given the calcium $\frac {\Delta F}{F}$ fluorescence signals which we obtained before, we used the algorithm in its original implementation as a module of the toolbox [36, 51] apart from minor adjustments in order to allow it to run unattended. In particular, referring to the steps outlined by [53], in Step 21, we set the imaging frequency to $\frac {1}{\Delta T}$ and the decay time constant to $\frac {\tau _{1/2}}{\ln {2}}$. In Step 25, we chose the Gaussian model to estimate the noise in the fluorescence signal. In Step 26, we chose the dynamic threshold to estimate the significance of fluorescence transients and, in Step 28, set the minimal confidence for significance to 95%. Finally, in Step 40, we chose PCA-promax as the clustering algorithm. In Steps 41 and 42, we automated the choice of the zMax-cut-off value by selecting the first local minimum of the distribution after smoothing it with 10,000 points and a half of the suggested standard bandwidth. In the rare case when this unattended procedure failed or yielded an unsatisfactory zMax-cut-off value, e.g. when the distribution did not have a local minimum, we made the selection manually.

#### Promax-CS algorithm

In the Promax-MP algorithm, the significant principal components were estimated employing the Marčenko-Pastur distribution as a null model. Alternatively and analogously to the ICA-CS algorithm, this could also be done using circular shifts. Thus, leaving the rest of the algorithm unchanged, we estimated the null distribution with 500 iterations of circular shifts to estimate the null distribution, thresholding at the 95th percentile using the same functions as in the ICA algorithm.

#### SGC algorithm

The SGC algorithm [15] takes a different approach from the algorithms described above, which are based on pairwise correlations between neurons. Instead, it groups frames based on their pairwise similarity, regarding similar frames as representations of the same assembly. This idea is formalised in a graph-theoretic setting, in which identifying assemblies becomes analogous to identifying community structure in a graph.

A set of (binary) activity patterns was obtained from the calcium imaging data by thresholding the calcium $\frac {\Delta F}{F}$ fluorescence signal for every neuron *n* at two standard deviations above the mean, yielding a binary indicator signal for the units’ activity over the course of the recording. The activity of the whole array at a time *t* is referred to as the activity pattern, given by the binary *N*-tuple in which every component represented the state of activity of a neuron. When no assemblies were active (i.e. solely in the presence of noise), these activity patterns tended to be rather sparse. Therefore, only activity patterns with an overall high activity potentially corresponding to assembly events were considered. These patterns were determined when the number of active units (i.e. coactivity level) exceeded a significance threshold. This threshold was estimated by permuting the binary signals for every neuron independently 1000 times as the 95th percentile of the coactivity level.

The high activity activity patterns {*x*_*τ*_}_*τ*_ were put into relation to each other on a graph where every edge represented similarity between the incident nodes. More precisely, distance between activity patterns was measured in terms of the cosine-distance $d_{\text {cosine}} ({x, x'}) = 1 - \frac {\langle {x}\,\ { x'}\rangle }{\parallel {x}\parallel \ \parallel {x'}\parallel }$ and the graph constructed as an unweighted *k*-nearest-neighbour graph. *k* was initially chosen to be equal to ⌈ln|{*x*_*τ*_}_*τ*_|⌉ [32]. In the unusual event that this led to a graph that was disconnected, *k* was increased in steps of 1 until the resulting graph was connected.

By construction, community structure in such a graph corresponded to groups of activity patterns which were more similar within each group than to other groups. Thus, in order to extract assemblies, the community structure within the graph needed to be identified. While there are many graph clustering algorithms, they generally all assume some prior knowledge of the number of communities, i.e. the expected number of assemblies. To estimate this number, we used a recently proposed approach based on statistical inference methods [54, 55]. Using this approach, a degree-corrected stochastic block model is fitted to the observed graph and from this fit the most likely number of communities present can be inferred. Given this number, the graph was decomposed into its communities using spectral clustering methods [32, 56], in order to arrive at a clustering of the activity patterns $\{\{x_{\tau _{r}}\}_{\tau _{r}}\}_{r}$.

Every group or community $\{x_{\tau _{r}}\}_{\tau _{r}}$ obtained from that consisted of a set of similar activity patterns, and we obtained assembly patterns by averaging over the patterns in each group. This resulted in continuous-valued components. Since the value for a neuron in this pattern was 1 when it was consistently active for all the patterns in a group and 0 when it was never active, the values within these assembly activity patterns were interpreted as the affinity of each neuron to the pattern.

However, we found that the results could be improved by disregarding activity patterns from averaging that were due merely to a high level of noise. In particular, we applied an optimisation procedure to reject certain activity patterns. Given the groups of activity patterns from the spectral clustering, first any group which consisted of too few activity patterns, i.e. less then 5, was rejected. Then, assuming that every assembly was active, a comparable number of times, every group which was smaller than one and a half standard deviations below the mean was rejected. From the remaining groups, we obtained a set of preliminary core assembly patterns by averaging the activity patterns and binarising the resulting mean pattern at a threshold value of $s = \frac {1}{5}$, $\hat {\alpha }_{r} = \mathbbm{1}_{[ s, \infty [}\left ({\overline {\{x_{\tau _{r}}\}_{\tau _{r}}}}\right)$, where the bar denotes averaging and the indicator function is applied component-wise to the average activity pattern. Groups were then merged if the similarity between their corresponding core assembly patterns exceeded a threshold, in particular

$$\min\left(\frac{\langle{\hat{\alpha}'}\,\ {\hat{\alpha}}\rangle}{\|{\hat{\alpha}}\|^{2}}, \frac{\langle{\hat{\alpha}}\,\ {\hat{\alpha}'}\rangle}{\|{\hat{\alpha}'}\|^{2} }\right) > p $$

for $\hat {\alpha }$ and $\hat {\alpha }'$ two distinct core assembly patterns with $p = \frac {2}{3}$. Doing this recursively gave potentially different groups and therefore a new set of preliminary core assembly patterns. The groups so far were disregarded and the preliminary core assembly patterns defined a new set of groups. Provided that an activity pattern *x* was similar enough to one of the preliminary core assembly patterns $\hat {\alpha }$, in the sense that the conditions

$$\frac{\langle{\hat{\alpha}}\, \ {x}\rangle}{\|{\hat{\alpha}}\|^{2}} > p \quad \text{and} \quad \frac{\|{x}\|^{2}}{\|{\hat{\alpha}}\|^{2}} > p $$

were satisfied with $p = \frac {1}{2}$, it was assigned to the group defined by the most similar preliminary core assembly pattern, otherwise it was rejected. This yielded a usually different and smaller clustering of the activity patterns of the form $\{\{x_{\tau _{r}}\}_{\tau _{r}}\}_{r}$. These conditions ensured that at least a fraction *p* of the activity pattern’s (active) neurons overlapped with those of the preliminary core assembly pattern and that at least a fraction *p* of the overall activity in the preliminary core assembly pattern was also present in the activity pattern.

After every activity pattern was assigned to a group defined by one of the preliminary core assembly patterns, or rejected, finally also all the activity patterns from groups whose size was smaller than one and a half standard deviations below the mean were rejected. The remaining groups defined the core assembly patterns, which were obtained by averaging the corresponding activity patterns, $\alpha _{r} = \overline {\{x_{\tau _{r}}\}_{\tau _{r}} }$, while the strength of affinity of every neuron to the assembly pattern was used to determine whether to assign the unit to an assembly by thresholding at an affinity level *s*.

#### CORE algorithm

Similar to the SGC algorithm, the CORE algorithm [7] also effectively groups frames on their pairwise similarity in order to recover the assemblies. In this case, first spike probabilities were inferred from the calcium $\frac {\Delta F}{F}$ fluorescence signal, applying fast, nonnegative deconvolution [33] of every neuron separately. In order to interpret the output as probability, it was normalised to a maximum value of 1 for every unit. The spike probabilities were then thresholded at three standard deviations from 0, yielding a binary indicator signal for the units’ activity over the course of the recording. As in the SGC algorithm, the activity of the whole array at time *t* is referred to as an activity pattern, given by a binary *N*-tuple, and only activity patterns with an overall high activity were considered and determined using the same permutation test as for SCG.

The activity patterns {*x*_*τ*_}_*τ*_ with an overall high level of coactivation, in that context also referred to as ensembles, were then compared to each other using Pearson’s correlation coefficient. The significance for each comparison was determined using a permutation test. The ensembles were permutated independently 50,000 times and the significance threshold was estimated as the 95th percentile. Given an ensemble *x*_*τ*_ and with [*x*_*τ*_] being the set of ensembles which are significantly similar to *x*_*τ*_, the corresponding core ensemble was defined as $\hat {x}_{\tau } = \mathbbm{1}_{[ s, \infty [}\left (\overline {[x_{\tau }]}\right)$ with a threshold value of $s = \frac {1}{2}$. While *s*=1 in the original description of the algorithm, we found this to be too restrictive, as the core ensembles turned out to be very sparse or even empty.

At this stage, we found that the set of core ensembles contained groups of highly similar frames, so in a final step, we clustered these core ensembles. We used k-means clustering with distance between any two core ensembles given by the Hamming distance. In particular, we chose the optimal number of clusters *k* which yielded the maximal Silhouette coefficient [57] in 1000 rounds of k-means clustering and obtained the final clustering after another 1000 rounds of k-means clustering for the optimal number of clusters *k* and optimising the Silhouette coefficient as $\{\{\hat {x}_{\tau _{r}}\}_{\tau _{r}}\}_{r}$. The core ensemble pattern were then defined as $\alpha _{r} = \overline {\{x_{\tau _{r}}\}_{\tau _{r}} }$, while the strength of affinity of every neuron to the pattern was used to determine whether to assign the unit to an assembly by thresholding at an affinity level *s*.

#### SVD algorithm

For the SVD algorithm [31], we used the recent implementation available at https://github.com/hanshuting/SVDEnsemble [58]. Here one starts by transforming the calcium $\frac {\Delta F}{F}$ fluorescence signal into a binary signal. As in the CORE algorithm spike probabilities were first inferred by applying fast, nonnegative deconvolution [33], and these probabilities were then thresholded. Only activity patterns with an overall high activity were considered. The threshold was determined using the 99th percentile of the same permutation test.

The activity patterns {*x*_*τ*_}_*τ*_ with an overall high level of coactivation were then normalised according to a term frequency-inverse document frequency (TF-IDF) algorithm in order to find the most relevant neurons. In that sense, the TF-IDF was a measure of importance of a specific neuron to the activity of the whole population. The TF-IDF-normalised activity patterns $\{\hat {x}_{\tau }\}_{\tau }$ gave rise to a matrix $\left ({s}_{\text {cosine}}(\hat {x}_{\tau }, \hat {x}_{\tau '})\right)_{\tau,\tau '}$, the similarity map in terms of the cosine similarity $s_{\text {cosine}}{(x, x')} = \frac { \langle {x }\,\ { x' }\rangle }{\|{x}\|\ \|{x'}\|}$.

This matrix of similarity between activity patterns was transformed into a binary matrix and the threshold was determined by a permutation test as the 98th percentile from 20 permutations. Afterwards, the binary similarity map was once more transformed into a matrix which held the pairwise similarities between columns (or rows) of the binary similarity map in terms of the Jaccard index and which was again thresholded using a permutation test. In order to identify the neuronal ensembles, a singular decomposition was obtained for this matrix and the number of significant singular values was inferred from the singular values above the chance level. Finally, the composition of the assemblies was determined from the activity patterns corresponding to every significant singular vector.

#### FIM-X algorithm

For this algorithm [29, 30], we used the implementation available at http://www.borgelt.net/python/psf+psr.zip[59]. Given the identity of every neuron which is active at every point in time, the technique of frequent item set mining [60, 61] was applied to find every item set of neurons which was frequent with a level *s*_min_, which were then considered candidates for assemblies. Closed frequent item sets only were considered.

In order to exclude item sets that were frequent simply by chance, shuffled datasets were analysed which were generated from the original data with the intention of preserving all essential features of the data, while destroying any synchrony. Applying frequent item set mining to these surrogates, all frequent item sets or rather their signatures were collected. An item set’s signature was its support, i.e. the number of instances where it appeared, together with its size. The signatures from many surrogate datasets were then used to discriminate informative frequent item sets in the original data from the rest by disregarding any frequent item set whose signature also appeared in the surrogate datasets (“pattern spectrum filtering” – PSF). The rationale for doing so was that any frequent item set, whose signature also occurred in surrogates, where synchrony was destroyed so that items could be essentially regarded as independent, cannot be informative in identifying an assembly. Finally, the remaining class of closed frequent item sets was statistically analysed (“pattern set reduction”—PSR). A pair of closed frequent item sets, where one is a subset of the other, was assessed for the conditional significance of one given that the other represents an assembly, and vice versa, in order to arrive at a class of item sets which were pairwise mutually significant when conditioned on each other. This class was then taken to represent the assemblies underlying the original data.

We applied this algorithm to the binary activity patterns which we obtained from thresholding every neuron’s $\frac {\Delta F}{F}$ fluorescence signal at two standard deviations above its mean. In particular, we used the implementation utilising frequent item set mining on discrete time data as opposed to its generalisation to continuous time data. While always considering only closed frequent item sets, we set the frequency threshold to *s*_min_=6. We generated 1000 surrogate datasets using the method of permutation by pair swaps and used the covered-spike criterion (*z*_*A*_−1)*c*_*A*_:(*z*_*B*_−1)*c*_*B*_ for the pattern set reduction.

### Measuring performance using Best Match score

While several measures have been proposed to evaluate the “difference” between two clusterings $\mathcal {A} = \left \{A_{1}, \ldots A_{|{\mathcal {A}}|}\right \}$ and $\mathcal {A}' = \left \{A'_{1}, \ldots A'_{|{\mathcal {A}'}|}\right \}$, the elements of every clustering were usually assumed to be pairwise non-intersecting [37, 62]. Hence, since in our simulated calcium activity we explicitly allowed for the possibility of overlapping assemblies, we measured differences of clusterings in terms of a “Best Match” distance [37], which was defined as

$${{\begin{aligned} \hspace{15pt} {\text{BestMatch}_{d}}(\mathcal{A}, \mathcal{A}') &= \sum\limits_{A \in \mathcal{A}} \min_{A' \in \mathcal{A}'} d{(A, A')} \\&+ \sum_{A' \in \mathcal{A}'} \min_{A \in \mathcal{A}} d(A', A){,} \end{aligned}}} $$

with the set difference measure $d{(A, A')} = 1 - \frac {|{A \cap A'}|}{|{A \cup A'}|}$ to measure the difference between two clusters *A* and *A*^′^ (as before, *A*∪*A*^′^ and *A*∩*A*^′^ denote the union and intersection of *A* and *A*^′^, and | · | for a set its cardinality). From that we defined the Best Match score as

$${{\begin{aligned} \hspace{39pt} &\mathrm{{BestMatch}_{d}-score}{\left(\mathcal{A}, \mathcal{A}'\right)} \\&= 1 - \frac{1}{|{\mathcal{A}}| + |{\mathcal{A}'}|} \text{BestMatch}_{d}{\left(\mathcal{A}, \mathcal{A}'\right)} \end{aligned}}} $$

to quantify the similarity between two clusterings.

To interpret the Best Match score, we needed to identify its chance level, i.e. the values expected when comparing two random clusterings. Therefore, we assumed a population of size *N* and two independent random variables *A* and *A*^′^ which took values uniformly in the subsets of {1,…*N*} with *N*_*A*_ and $N_{A'}\phantom {\dot {i}\!}$ elements respectively, where *N*_*A*_ and $\phantom {\dot {i}\!}N_{A'}$ are random variables independent of each other and of *A* and *A*^′^. Then, since the number of elements in the intersection of *A* and *A*^′^ is hypergeometrically distributed, $\phantom {\dot {i}\!}|{A \cap A' }\| N_{A}, N_{A'} \sim \text {Hypergeom}_{N;N_{A},N_{A'}}$, we obtained

$$\begin{aligned} \mathbb{P}[ d{\left(A,A'\right)} = u ] = \sum_{n_{A},n_{A'} = 1}^{N} \mathbb{P}[ N_{A} = n_{A} ] \mathbb{P}[ N_{A'} = n_{A'} ]\\ \times \text{Hypergeom}_{N;n_{A},n_{A'}}{\left({(n_{A} + n_{A'})} {\left(\frac{1 - u}{2 - u} \right)}\right)} {.} \end{aligned} $$

If we assumed a collection $\mathcal {A}'$ of independent and identically distributed copies of *A*^′^, we had

$${{\begin{aligned} \hspace{15pt} \mathbb{P}\left[ \min_{A' \in \mathcal{A}'} d{\left(A,A'\right)} = u \right] =& \mathbb{P}[ d{\left(A,A'\right)} \geq u ]^{|{\mathcal{A}' }|}\\ &- \mathbb{P}[ d{\left(A,A'\right)} > u ]^{|{\mathcal{A}'}|} \end{aligned}}} $$

so that, by the linearity of the expectation,

$$\begin{aligned} &\mathbb{E}[\text{BestMatch}_{d}-\text{score}{(\mathcal{A}, \mathcal{A}')} ] = 1 - \frac{1}{|{\mathcal{A}}| + |{\mathcal{A}'}|}\\ &\quad \times\left({|{\mathcal{A}|} \mathbb{E}{\left[\min_{A' \in \mathcal{A}'} d{(A,A')}\right]} + |{\mathcal{A}'}| \mathbb{E}{\left[\min_{A \in \mathcal{A}} d{(A',A)}\right]} }\right) {,} \end{aligned} $$

where $\phantom {\dot {i}\!}\mathbb {E}{\left [\min _{A' \in \mathcal {A}'} d{\left (A,A'\right)}\right ]}$ could be computed from its distribution given above, as well as also $\phantom {\dot {i}\!}\mathbb {E}{\left [\min _{A \in \mathcal {A}} d{\left (A',A\right)}\right ]}$ after reversing the roles of *A* and *A*^′^.

Since the Best Match score compared two clustering as a whole, it reported low similarity even if one clustering was essentially a subset of the other. Thus, given a reference clustering $\mathcal {A}$ we were interested in the “optimal Best Match score” as the Best Match score between this clustering and some sub-clustering of $\mathcal {A}'$, provided that the latter was a larger clustering. Following a greedy algorithm, we extracted such a sub-clustering iteratively, selecting in every round pairs (*A*,*A*^′^) from $\mathcal {A} \times \mathcal {A}'$ such that the choice was optimal in the sense that the distance between the two sets was minimal. More precisely, in the *r*th round the pair selected was

$${\left(A_{r}, A'_{r}\right)} = \underset{(A,A') \in {(\mathcal{A} \setminus \{A_{1},\ldots A_{r-1}\})} \times {\left(\mathcal{A}' \setminus \{A'_{1},\ldots A'_{r-1}\}\right)}}{\mathrm{arg\,min}} d{\left(A,A'\right)} {.} $$

In case this choice was not unique, a pair was selected randomly among those satisfying the requirements. This iterative procedure terminated after round $R = \min {\left (|{\mathcal {A}|}, |{\mathcal {A}'}|\right) }$ and the optimal sub-clustering of $\mathcal {A}'$ relative to $\mathcal {A}$ was given as $\mathcal {A}'_{*} = \left \{A'_{1}, \ldots A'_{R}\right \}$. Thus we defined the optimal Best Match score between $\mathcal {A}$ and $\mathcal {A}'$ as the Best Match score between $\mathcal {A}$ and $\mathcal {A}'_{*}$.

Although $\mathcal {A}'_{*}$ was introduced as the optimal sub-clustering, it does not necessarily satisfy the relation that $\text {BestMatch}_{d}\,-\,\text {score}{(\!\mathcal {A},\\ \mathcal {A}')} \leq \text {BestMatch}_{d}\,-\,\text {score}{(\mathcal {A}, \mathcal {A}'_{*}) }$, due to the normalisation factor. However, in practice the optimal sub-clustering $\mathcal {A}'_{*}$ provided in most cases a better match with respect to $\mathcal {A}$ than $\mathcal {A}'$.

### Computational procedures

All simulations and analysis was performed on two of The University of Queensland’s high-performance computing clusters. For every parameter variation, 400 datasets of surrogate calcium imaging data were simulated and analysed. The implementation was predominately done in MATLAB (MathWorks) and is available as a GitHub repository, at https://github.com/GoodhillLab/neural-assembly-detection.

The benchmark measurements were all conducted on a cluster where each computing node was a Dell EMC PowerEdge R740 server comprised of 2 Intel Xeon Gold 6132 processors with 14 cores at a base frequency of 2.60 GHz each, 384 GB DDR4 RAM, and 1.6 TB Dell EMC NVMe flash strorage. The benchmark measurements were allocated 1 CPU core and 16 GB of RAM each and output was written to the flash storage.

### Experimental procedures

All procedures were performed with approval from The University of Queensland Animal Ethics Committee. *Nacre* zebrafish (*Danio rerio*) embryos expressing *elavl3:H2B-GCaMP6s* of either sex were collected and raised according to established procedures [63] and kept under a 14/10 h on/off light cycle. Larvae were fed rotifers (*Brachionus plicatilis*) from 5 dpf (days postfertilization). Nine dpf zebrafish larvae were embedded in 2% low-melting point agarose in E3 embryo medium in a 35 mm plastic petri dish and the agarose was overlaid with E3. Calcium imaging was performed at a depth of 60 *μ*m from the dorsal surface of the tectal midline using a Zeiss LSM 710 2-photon microscope equipped with a Zeiss 40 ×/1.0 NA water-dipping objective. The sample was excited via a Spectra-Physics Mai Tai DeepSee Ti:Sapphire laser (Spectra-Physics) at an excitation wavelength of 940 nm and sampled at 2.2 Hz.

Visual stimuli were generated using MATLAB (MathWorks) and the Psychophysics Toolbox, and consisted of 6-degree-wide black spots at 11 different azimuth positions, which were presented for 1 s each, followed by 15 s of blank screen to allow calcium signals to return to baseline levels. Eleven spots trials were presented 20 times with 25 s of inter-trial interval. 90° azimuth was defined as being orthogonal to the body axis at the eye. Visual stimuli were projected onto the wall of the dish using an Optoma PK302 mini-projector and covered 150° of the visual field, from 15° to 165°. To synchronise image acquisition and the delivery of visual stimuli, we used a NA-USB-6501 I/O TTL device. Subsequent image processing and extraction of neural activity was performed as in [15].

#### Estimation of a reference assembly configuration

Given the knowledge about the stimulus presentation times, we reconstructed the underlying, stimulus-evoked reference assembly configuration by examining the average population response to every stimulus presentation. In particular, we obtained the average population response for every stimulus by averaging the amplitude of neural traces in frames 3–7 after each stimulus onset. We then considered a neuron as part of the corresponding stimulus-evoked assembly if its average fluorescence exceeded *p* standard deviation from the mean across average responses to all stimuli. This defined the *p*th reference assembly configuration.

## Additional files

Additional file 1 Performance as a function of assembly overlap and the saturation constant. Graphing conventions as in Fig. 3. **A**, **B:** Varying the assembly overlap. With increasing overlap, CORE detects a decreasing number of assemblies, while for ICA-CS, ICA-MP and Promax-CS this is only a slight decrease and, consequently, their performance decreased. SGC, Promax-MP and FIM-X detected a constant number of assemblies, but Promax-MP and FIM-X overestimated the number of assemblies. **C**, **D:** Varying the saturation constant *κ*. For all algorithms the number of detected assemblies and the overall performance was approximately constant. (PDF 231 kb)

Additional file 2Optimal performance (Optimal Best Match score) as a function of all the varied parameters. Graphing conventions as in Fig. 3 for the performance graphs. **A:** Varying the size of the neural array (cf. Fig. 3a, b). ICA-CS, ICA-MP and SGC showed good optimal performance. The optimal performance of all algorithms was approximately constant. **B:** Varying the number of embedded assemblies (cf. Fig. 3c, d). ICA-CS, ICA-MP and SGC showed good optimal performance. When increasing the number of assemblies, Promax-CS showed an increase in optimal performance. The optimal performance of all other algorithms was approximately constant. **C:** Varying the assembly sizes (cf. Fig. 3e, f). When increasing the assembly size, the optimal performance of ICA-CS, ICA-MP, SGC and CORE increased to good performance. While Promax-MP showed good optimal performance for small assembly sizes, Promax-CS showed it over the whole range. **D:** Varying the assembly overlap (cf. Additional file 1: Figure S1A,B). When increasing the overlap, the optimal performance of all algorithms except SGC decreased. **E:** Varying the simulation duration *T* (cf. Fig. 4a, b). With increasing *T*, the performance of ICA-CS, ICA-MP, SGC and Promax-CS increased to good optimal performance beyond *T*=1800*s*. **F:** Varying the temporal resolution *ΔT* (cf. Fig. 4c, d). ICA-CS, ICA-MP, Promax-CS and SGC showed good optimal performance. The optimal performance of all algorithms was approximately constant. **G:** Varying the calcium indicator half-life $\tau _{\frac {1}{2}}$ (cf. Fig. 4e, f). With increasing $\tau _{\frac {1}{2}}$, all algorithms showed a decrease in optimal performance. ICA-CS, ICA-MP, SGC and Promax-CS showed good optimal performance. **H:** Varying the event frequency *f*^∗^ (cf. Fig. 5a, b). With increasing *f*^∗^, the optimal performance of all algorithms increased. ICA-CS, ICA-MP, SGC and Promax-CS reached good optimal performance at large *f*^∗^. **I:** Varying the event firing rate multiplier *λ* (cf. Fig. 5c, d). With increasing *λ*, the performance of ICA-CS, ICA-MP, SGC, Promax-MP, Promax-CS and FIM-X increased and reached good optimal performance at large *λ*. After an initial increase the performance of CORE decreased towards large *λ*. **J:** Varying the standard deviation *σ* of the Gaussian noise (cf. Fig. 5e, f). With increasing *σ*, the optimal performance of every algorithm decreased. For noise levels beyond *σ*=3 neither Promax-MP or Promax-CS yielded any results as they were not able to fit the noise model to the data. **K:** Varying the saturation constant *κ* (cf. Fig. S1c, d). ICA-CS, ICA-MP and SGC showed good optimal performance. The optimal performance of all algorithms was approximately constant. (PDF 561 kb)

Additional file 3 Performance as a function of the simulation duration and event frequency, rescaled in terms of the average number of activations per assembly. Graphing conventions as in Fig. 3. **A**, **B:** Varying the simulation duration *T* (cf. Fig. 4a, b), rescaled as varying the average number of activations per assembly. **C**, **D:** Varying the event frequency *f*^∗^ (cf. Fig. 5a, b), rescaled as varying the average number of activations per assembly. Notably variations in the simulation duration yield slightly different results than variations in the event frequency. (PDF 219 kb)

Additional file 4 Assembly detection performance for the estimated reference assembly configuration. The performance of the different algorithms as a function of the parameter *p* of the reference assembly configuration. For most values of *p* SGC performed best, apart from large values of *p* where all algorithms performed poorly. (PDF 80.7 kb)

Additional file 5 Runtime (walltime) as a function of the size of the neural array and the simulation duration. In every graph and for every algorithm the mean is depicted by a solid line together with the region of one standard deviation above and below. The measurements were conducted on a high performance computing cluster, where for the analysis of every dataset 1 CPU core and 16 GB of RAM were allocated and output was written to the flash storage. The relative runtime for every algorithm was computed relative to the runtime at the default parameters (cf. Table. 1). **A**, **B:** Varying the size of the neural array. **C**, **D:** Varying the simulation duration *T*. (PDF 189 kb)
