# Correction to: Detecting neural assemblies in calcium imaging data

**DOI:** 10.1186/s12915-019-0644-6

**Published:** 2019-03-06

**Authors:** Jan Mölter, Lilach Avitan, Geoffrey J. Goodhill

**Affiliations:** 1 Queensland Brain Institute, The University of Queensland, Brisbane, 4072 Australia; 2 School of Mathematics and Physics, The University of Queensland, Brisbane, 4072 Australia

**Correction to: BMC Biol**

**https://doi.org/10.1186/s12915-018-0606-4**

Upon publication of the original article, [1], the authors noticed that the first authors’ affiliation contained an error. In the first affiliation it reads “Queensland Brian Institute”, whereas it should in fact be “Queensland Brain Institute”.
