# Supplementary figures and images for: Detecting neural assemblies in calcium imaging data
Source: BMC Biol. 2018 Nov 28;16:143. doi: 10.1186/s12915-018-0606-4 (PMC6262979; doi:10.1186/s12915-018-0606-4)

**A**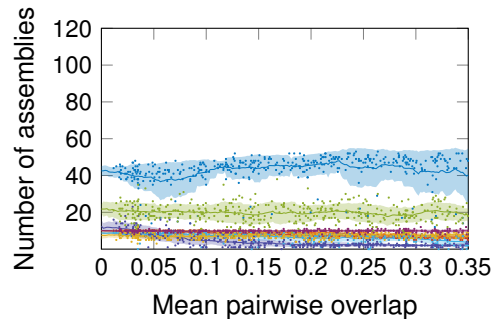**B**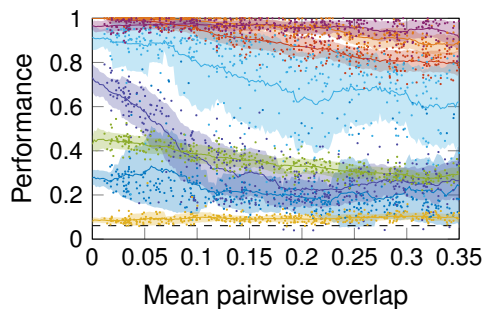**C**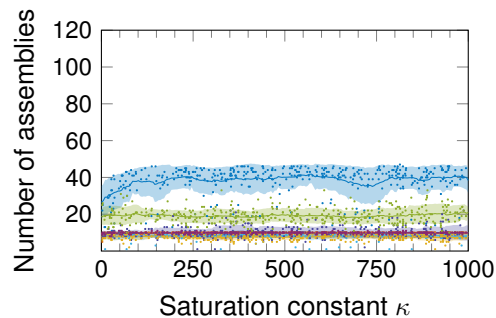**D**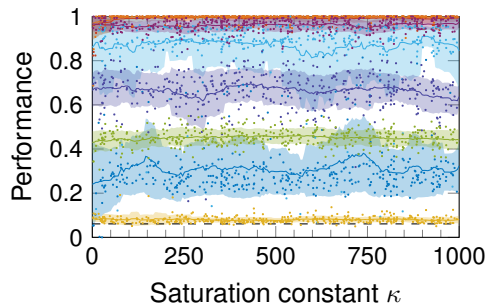

**Algorithm:** ● ICA-CS | ● Promax-MP | ● CORE | ● SVD | ● SGC | ● FIM-X  
● ICA-MP | ● Promax-CS

Supplement: Supplementary file 1 — Performance as a function of assembly overlap and the saturation constant. Graphing conventions as in Fig. 3. A, B: Varying the assembly overlap. With increasing overlap, CORE detects a decreasing number of assemblies, while for ICA-CS, ICA-MP and Promax-CS this is only a slight decrease and, consequently, their performance decreased. SGC, Promax-MP and FIM-X detected a constant number of assemblies, but Promax-MP and FIM-X overestimated the number of assemblies. C, D: Varying the saturation constant κ. For all algorithms the number of detected assemblies and the overall performance was approximately constant. (PDF 231 kb) [file 12915_2018_606_MOESM1_ESM.pdf]

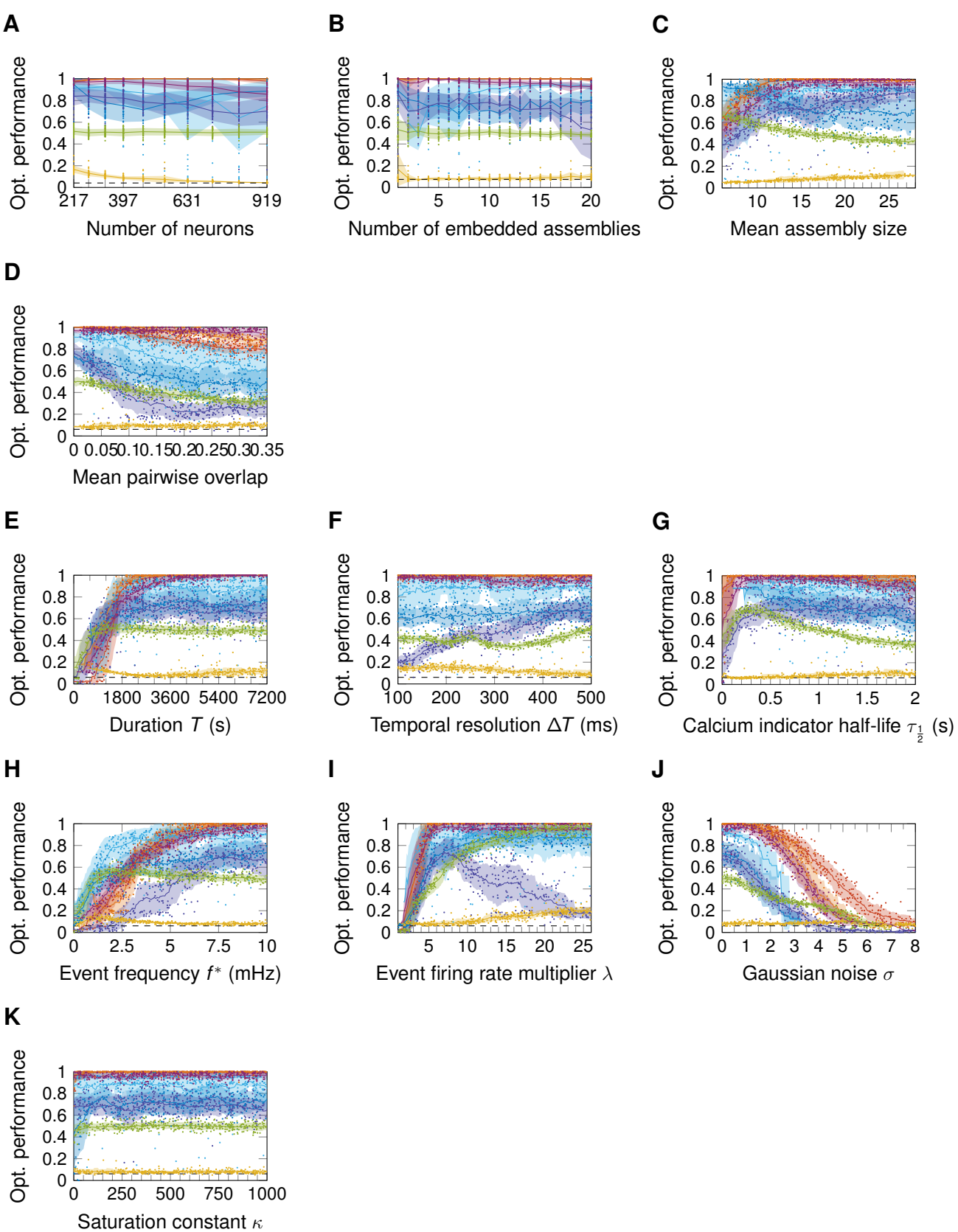

**Algorithm:** ● ICA-CS | ● Promax-MP | ● CORE | ● SVD | ● SGC | ● FIM-X  
 ● ICA-MP | ● Promax-CS

Supplement: Supplementary file 2 — Optimal performance (Optimal Best Match score) as a function of all the varied parameters. Graphing conventions as in Fig. 3 for the performance graphs. A: Varying the size of the neural array (cf. Fig. 3a, b). ICA-CS, ICA-MP and SGC showed good optimal performance. The optimal performance of all algorithms was approximately constant. B: Varying the number of embedded assemblies (cf. Fig. 3c, d). ICA-CS, ICA-MP and SGC showed good optimal performance. When increasing the number of assemblies, Promax-CS showed an increase in optimal performance. The optimal performance of all other algorithms was approximately constant. C: Varying the assembly sizes (cf. Fig. 3e, f). When increasing the assembly size, the optimal performance of ICA-CS, ICA-MP, SGC and CORE increased to good performance. While Promax-MP showed good optimal performance for small assembly sizes, Promax-CS showed it over the whole range. D: Varying the assembly overlap (cf. Additional file 1: Figure S1A,B). When increasing the overlap, the optimal performance of all algorithms except SGC decreased. E: Varying the simulation duration T (cf. Fig. 4a, b). With increasing T, the performance of ICA-CS, ICA-MP, SGC and Promax-CS increased to good optimal performance beyond T=1800s. F: Varying the temporal resolution ΔT (cf. Fig. 4c, d). ICA-CS, ICA-MP, Promax-CS and SGC showed good optimal performance. The optimal performance of all algorithms was approximately constant. G: Varying the calcium indicator half-life $\tau _{\frac {1}{2}}$τ12 (cf. Fig. 4e, f). With increasing [file 12915_2018_606_MOESM2_ESM.pdf]

**A**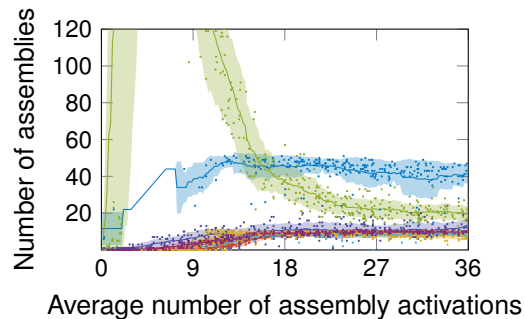**B**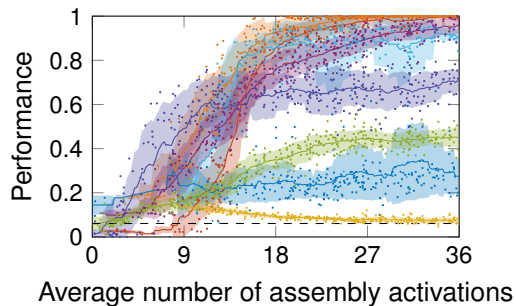**C**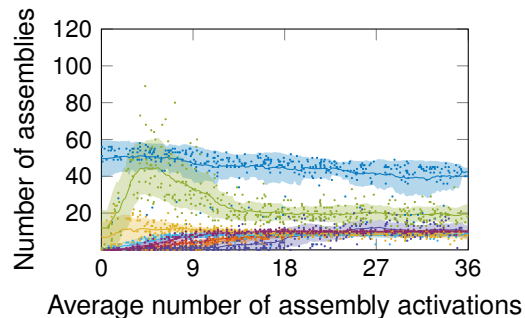**D**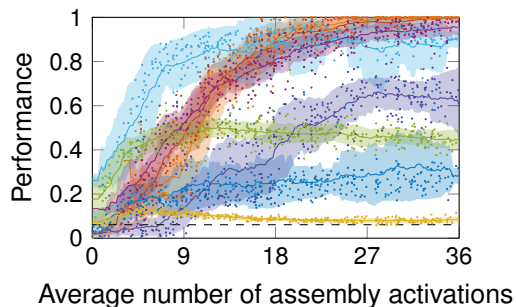

**Algorithm:** ● ICA-CS | ● Promax-MP | ● CORE | ● SVD | ● SGC | ● FIM-X  
● ICA-MP | ● Promax-CS

Supplement: Supplementary file 3 — Performance as a function of the simulation duration and event frequency, rescaled in terms of the average number of activations per assembly. Graphing conventions as in Fig. 3. A, B: Varying the simulation duration T (cf. Fig. 4a, b), rescaled as varying the average number of activations per assembly. C, D: Varying the event frequency f∗ (cf. Fig. 5a, b), rescaled as varying the average number of activations per assembly. Notably variations in the simulation duration yield slightly different results than variations in the event frequency. (PDF 219 kb) [file 12915_2018_606_MOESM3_ESM.pdf]

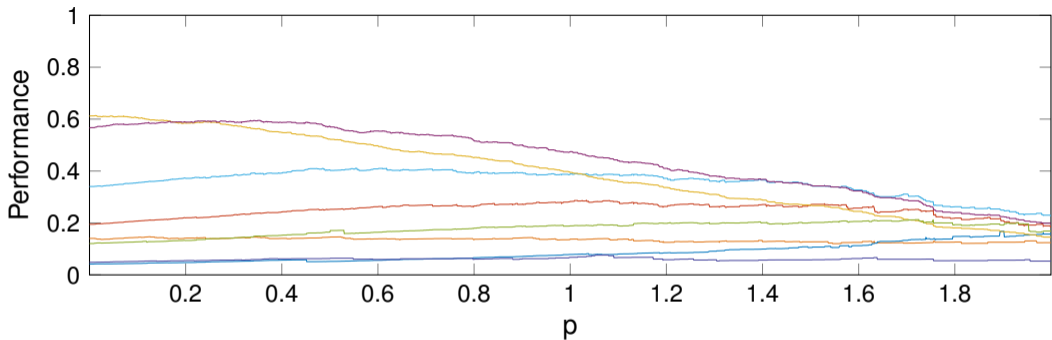

**Algorithm:** ● ICA-CS | ● Promax-MP | ● CORE | ● SVD | ● SGC | ● FIM-X  
● ICA-MP | ● Promax-CS

Supplement: Supplementary file 4 — Assembly detection performance for the estimated reference assembly configuration. The performance of the different algorithms as a function of the parameter p of the reference assembly configuration. For most values of p SGC performed best, apart from large values of p where all algorithms performed poorly. (PDF 80.7 kb) [file 12915_2018_606_MOESM4_ESM.pdf]

**A**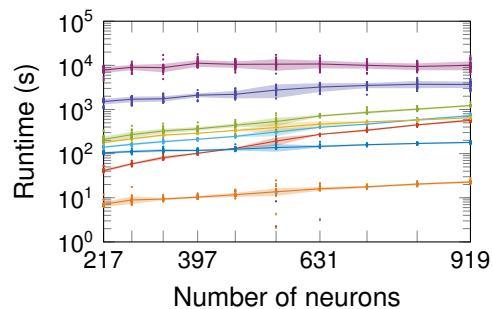**B**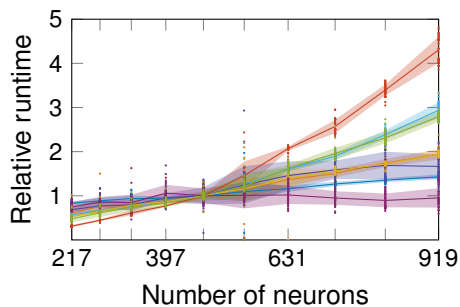**C**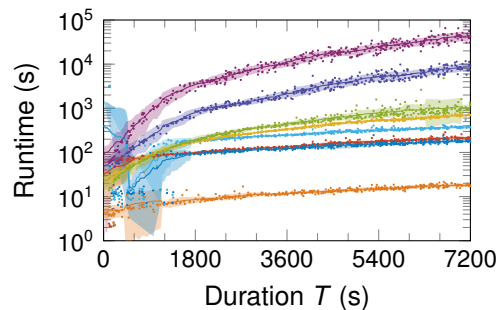**D**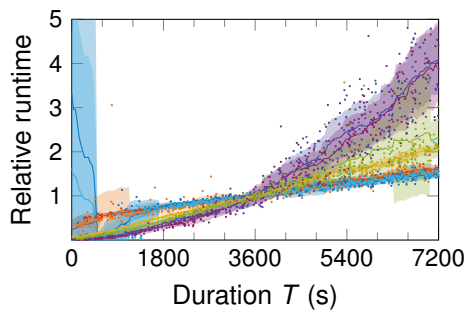

**Algorithm:** ● ICA-CS | ● Promax-MP | ● CORE | ● SVD | ● SGC | ● FIM-X  
● ICA-MP | ● Promax-CS

Supplement: Supplementary file 5 — Runtime (walltime) as a function of the size of the neural array and the simulation duration. In every graph and for every algorithm the mean is depicted by a solid line together with the region of one standard deviation above and below. The measurements were conducted on a high performance computing cluster, where for the analysis of every dataset 1 CPU core and 16 GB of RAM were allocated and output was written to the flash storage. The relative runtime for every algorithm was computed relative to the runtime at the default parameters (cf. Table. 1). A, B: Varying the size of the neural array. C, D: Varying the simulation duration T. (PDF 189 kb) [file 12915_2018_606_MOESM5_ESM.pdf]
